# Lyophilization for Formulation Optimization of Drug-Loaded Thermoresponsive Polyelectrolyte Complex Nanogels from Functionalized Hyaluronic Acid

**DOI:** 10.3390/pharmaceutics15030929

**Published:** 2023-03-13

**Authors:** Huu Van Le, Virginie Dulong, Luc Picton, Didier Le Cerf

**Affiliations:** Sciences & Technic Faculty, Univ Rouen Normandie, INSA Rouen Normandie, CNRS, PBS UMR 6270, 76000 Rouen, France

**Keywords:** lyophilization, nanogel, thermoresponsive, polyelectrolyte complex, hyaluronic acid, Jeffamine M-2005

## Abstract

The lyophilization of nanogels is practical not only for their long-term conservation but also for adjusting their concentration and dispersant type during reconstitution for different applications. However, lyophilization strategies must be adapted to each kind of nanoformulation in order to minimize aggregation after reconstitution. In this work, the effects of formulation aspects (i.e., charge ratio, polymer concentration, thermoresponsive grafts, polycation type, cryoprotectant type, and concentration) on particle integrity after lyophilization and reconstitution for different types of polyelectrolyte complex nanogels (PEC-NGs) from hyaluronic acid (HA) were investigated. The main objective was to find the best approach for freeze-drying thermoresponsive PEC-NGs from Jeffamine-M-2005-functionalized HA, which has recently been developed as a potential platform for drug delivery. It was found that freeze-drying PEC-NG suspensions prepared at a relatively low polymer concentration of 0.2 g.L^−1^ with 0.2% (m/v) trehalose as a cryoprotectant allow the homogeneous redispersion of PEC-NGs when concentrated at 1 g.L^−1^ upon reconstitution in PBS without important aggregation (i.e., average particle size remaining under 350 nm), which could be applied to concentrate curcumin (CUR)-loaded PEC-NGs for optimizing CUR content. The thermoresponsive release of CUR from such concentrated PEC-NGs was also reverified, which showed a minor effect of freeze-drying on the drug release profile.

## 1. Introduction

**Figure 1:**
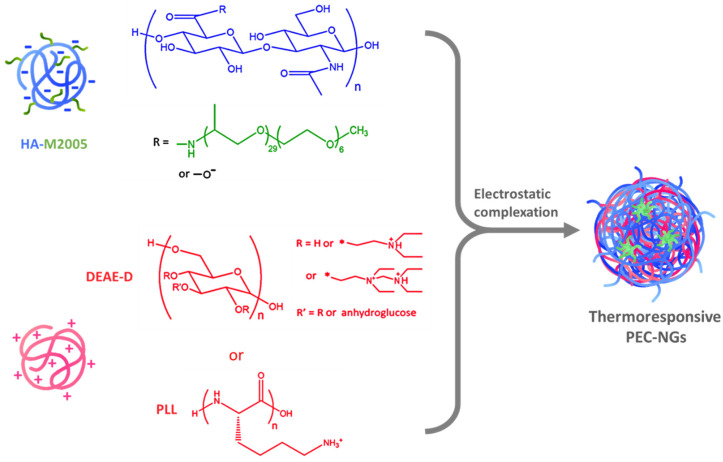
Thermoresponsive NGs formed by electrostatic interactions between HA-M2005 and polycations.

Nanogels (NGs) from polyelectrolyte complexes (PECs) of hyaluronic acid (HA) have been extensively studied in recent years as biomedical platforms with high potential that have the advantages of nanomedicine, green chemistry, and the inherent properties of HA [1]. Meanwhile, thermoresponsive nanocarriers from thermosensitive polymers exhibiting a lower critical solution temperature (LCST), i.e., increased hydrophobicity upon temperature increase, have also received great attention over the last two decades for drug delivery applications owing to their interesting behaviors, notably thermo-triggered drug release which can be controlled spatiotemporally [2,3]. Combining the two above-mentioned concepts, our previous work developed thermoresponsive polyelectrolyte complex nanogels (PEC-NGs) from the complexation between diethylaminoethyl dextran (DEAE-D) or poly-L-lysine (PLL) and hyaluronic acid grafted with Jeffamine M-2005 (M2005), i.e., thermoresponsive poly(ethylene oxide)-*co*-poly(propylene oxide) side-chains (Figure 1) [4]. Such PEC-NGs showed promising properties as nanocarriers for drug delivery, namely simple preparation, hyaluronidase-responsive degradation, thermo-controllable encapsulation of curcumin (CUR) as a model hydrophobic drug, and, especially, high stability in physiological saline media (PBS 1X at pH = 7.4) when including PLL as the constituent polycation [4]. With HA being the main component, these nanocarriers should also present good biocompatibility and the ability to target CD44 receptors on tumor cells [5]. However, their long-term storage stability and drug concentration remain to be optimized before further studies, e.g., drug release and biological assays [4].

In the above-mentioned context, lyophilization seems to be the most appropriate approach for optimizing both the aspects of conservation and formulation of the PEC-NGs. As reported in our previous work, PEC-NGs from HA-M2005 showed that particle size gradually increased over one month of storage in aqueous suspensions, probably due to the hydrophobic aggregation caused by M2005 chains on the particle’s surface [4]. Furthermore, the thermosensitivity of PEC-NGs in aqueous media, which can cause particle shrinkage or reswelling upon heating or cooling, respectively, would probably destabilize these PEC-NGs upon temperature variation during their transportation. In addition, aqueous media may favor microbiological contamination, which can cause HA degradation due to bacterial enzymes [6]. Therefore, water removal by freeze-drying has become a classical approach for better long-term conservation of colloidal systems [7]. Drying can also reduce product weight and volume to facilitate their storage and transportation. Moreover, the dried products after lyophilization may be dispersible within a freely adjustable volume of aqueous media to reach the desired concentration before their use [8]. The dispersant type can also be modified in this process, e.g., saline buffers of different physiological pH values for intended in vitro bioassays or in vivo applications.

Despite such practicality, lyophilization usually brings about irreversible particle aggregation due to different types of stress during the freezing phase, including ice crystal formation, cryoconcentration, and dehydration at the microstructure level [9,10]. The risk of destabilization during the drastic temperature decrease upon freezing may be even higher in the case of thermosensitive systems. For this reason, nanoparticles are usually freeze-dried in the presence of cryoprotectants, i.e., chemical agents which can stabilize the microstructure of the products during freezing [7]. Despite the importance of these aspects, few works have thoroughly described the lyophilization of HA-based PEC-NGs. The most relevant work of this type, to the best of our knowledge, was carried out by Umerska et al., who studied the effects of PEC composition and cryoprotectants on the particle integrity of PEC-NGs from chitosan (CTS) or protamine (PROT) in complex with HA or chondroitin sulfate after freeze-drying and reconstitution, for which poly(ethylene glycol) (PEG) and/or trehalose (TRE) were chosen as cryoprotectants [11]. In another work, Gheran et al. freeze-dried NGs from HA and CTS with glucose being selected as a cryoprotectant [12]. It can be seen that the lyophilization method should be adapted to each kind of nanoformulation to ensure particle stability but has not always been thoroughly explained and justified. In this context, the present work aims at understanding the impacts of formulation factors on particle integrity after the freeze-drying and reconstitution of PEC-NGs from HA or HA-M2005 in complex with DEAE-D or PLL, focusing on finding the best lyophilization strategy for HA-M2005/PLL PEC-NGs. The thermo-triggered drug release in vitro from the thermoresponsive PEC-NGs was also verified and reevaluated after lyophilization in order to evaluate the impact of this treatment on drug release.

## 2. Materials and Methods

### 2.1. Materials

HA with a weight-average molecular weight (M_w_) of 270,000 g.mol^−1^ was kindly offered by Givaudan (Pomacle, France). HA-M2005 obtained by grafting Jeffamine M-2005 (Huntsman, Houston, TX, USA) on HA with a degree of substitution of around 3.6% was retrieved from our previous work [4]. DEAE-D hydrochloride (M_w_ of 700,000 g.mol^−1^), α-PLL hydrobromide (M_w_ of 490,000 g.mol^−1^), CUR, phosphate-buffered saline (PBS) tablets, Tween 20, trehalose dihydrate, sucrose, glucose, and PEGs having number-average molecular weights (M_n_) of 2000; 10,000 and 20,000 g.mol^−1^ were purchased from Sigma-Aldrich (Saint-Quentin-Fallavier, France). Ascorbic acid was purchased from Acros Organics (Schwert, Seitingen-Oberflacht, Germany). Water used for all experiments was purified by the Milli-Q system (Millipore, Burlington, MA, USA).

### 2.2. Preparation of Blank PEC-NGs

PEC-NGs of predetermined charge ratios n−/n+ between HA or HA-M2005 as polyanions and DEAE-D or PLL as polycations were prepared as described in our previous work [4]. Briefly, polyanion and polycation solutions at defined polymer concentration (C_P_ = 0.1, 0.2, or 0.5 g.L^−1^) were prepared and filtered through regenerated cellulose 0.45 μm membrane filter units (Sartorius, Göttingen, Germany). To obtain PEC-NG suspensions at a predetermined C_P_, the polyanion solutions were rapidly dropped (one-shot) into the polycation solutions at the same C_P_, followed by mixing under mild stirring (200 rpm) at room temperature for 30 min. All samples were prepared, at least, in triplicate.

### 2.3. Curcumin Loading and Quantification in PEC-NGs

CUR-loaded HA-M2005/PLL PEC-NGs were prepared with the same protocol as our previous work [4]: an amount of 5 mg.mL^−1^ CUR stock solution in acetone was added to the HA-M2005 solution (C_P_ = 0.5 or 0.2 g.L^−1^), preheated at 50 °C, followed by rapid addition of PLL solution of the same C_P_ and temperature. The CUR to total polyelectrolyte mass ratio was 2:5. The mixture was then mildly stirred (200 rpm) at 50 °C for 30 min and, thereafter, left at room temperature for over 12 h for acetone evaporation. In order to remove precipitated CUR in excess and obtain homogenous PEC-NG suspensions, the mixture was centrifuged at 3000× *g* for 15 min and the supernatant was filtered through polyvinylidene difluoride (PVDF) 0.45 μm membrane filter units (Millipore, Burlington, MA, USA). All samples were prepared, at least, in triplicate and kept away from light to avoid CUR degradation.

The CUR encapsulated in PEC-NGs was quantified as described in our previous work [4]. Briefly, CUR-loaded PEC-NG suspensions were vortexed with HCl 1 N solution (PEC-NG suspension: HCl 1 N = 10:1 *v*/*v*), then with ethanol (aqueous phase: ethanol = 1:5 *v*/*v*). The mixture was then centrifuged at 3000× *g* for 15 min. The absorbance at λ = 420 nm of the supernatant was measured using a Cary 100 spectrophotometer (Agilent Technology, Santa Clara, CA, USA) to determine the CUR concentration in the supernatant and then the CUR concentration in the starting PEC-NG suspensions (C_CUR/SUS_). CUR loading capacity (LC) and encapsulation efficiency (EE) were estimated using Equations (1) and (2), with C_CUR/WATER_ being CUR solubility in Milli-Q water (0.3 μg.mL^−1^) [4], C_P_ being polymer concentration, and C_CUR0_ being the concentration of CUR initially added with respect to the aqueous phase.

LC = (C_CUR/SUS_ − C_CUR/WATER_)/C_P_ × 100 (%) (1)

EE = (C_CUR/SUS_ − C_CUR/WATER_)/C_CUR0_ × 100 (%) (2)

### 2.4. Particle Characterizations

The mean hydrodynamic diameter (D_h_) and polydispersity index (PDI) of PEC-NGs were determined by dynamic light scattering (DLS) technique in back-scatter mode using Zetasizer Ultra (Malvern Panalytical, Worcester, UK) for homogenous PEC-NG suspension samples without further dilution. Zeta potential (ζ) was measured by electrophoretic light scattering (ELS) technique with the same device. The temperature for all measurements was 25 °C with 120 s of temperature stabilization. All samples were measured, at least, in triplicate.

Particle morphology was verified by transmission electron microscopy (TEM). Specimens were prepared by placing a 10 μL droplet of PEC-NG suspension on a formvar-carbon-coated copper grid for 15 min. The droplet was then gently wicked away using a piece of filter paper. A 10 μL droplet of phosphotungstic acid 1% solution (contrast enhancer) was, thereafter, placed on the grid and removed after 30 s. The specimens were then air-dried and observed under an FEI Tecnai 12 BioTwin electron microscope (Phillips, Netherlands).

### 2.5. Freeze-Drying and Reconstitution of PEC-NGs

In the case of lyophilization with cryoprotectants, the cryoprotectants of interest (sucrose, glucose, trehalose, or PEGs) were dissolved at determined concentrations in PEC-NG suspensions by vortex mixing in 30 s. The PEC-NG suspensions, with or without cryoprotectants, were frozen overnight at −20 °C in a freezer. The samples were, thereafter, freeze-dried using an Alpha 1–2 LD Plus freeze-drier (Martin Christ, Germany) in primary drying mode for 24 h with pressure under 0.1 mbar, and the condenser temperature was −60 °C. To reconstitute PEC-NG suspensions, unless otherwise indicated, the freeze-dried PEC-NGs were dispersed by vortex mixing for 60 s in the same volume of Milli-Q water as before freeze-drying. Only visually homogeneous reconstituted suspensions were then subjected to DLS and/or ELS for particle characterization. For certain studies, different volumes of PBS 1X buffer (pH 7.4) were applied as dispersants during the reconstitution step according to the experiment’s purpose.

### 2.6. Curcumin Release In Vitro

CUR release from PEC-NGs was studied using the dialysis method adapted from the literature [13,14]. In brief, 3 mL of PEC-NG suspension was placed in a dialysis tube (D-Tube Dialyzer Maxi, MWCO 12–14 kDa, Millipore, Germany) and then immersed in 21 mL of PBS 1X (pH 7.4), with 0.5% (m/v) Tween 20 to ensure sink conditions and 0.5 mmol.L^−1^ ascorbic acid to avoid CUR oxidation. The dialysis was realized under mild stirring (100 rpm) at either 37 or 42 °C to investigate the impact of temperature. In order to quantify the cumulative fraction of CUR released, 700 μL of external media was taken out for CUR quantification and replaced by the same volume of fresh media at predetermined time points. CUR quantification was realized by measuring the absorbance at λ = 420 nm with a Cary 100 spectrophotometer (Agilent Technology, Santa Clara, MA, USA) and a standard curve of CUR concentration in the range of 0.01–1 μg.L^−1^ in the release medium (PBS 1X at pH = 7.4 with 0.5% m/v Tween 20 and 0.5 mmol.L^−1^ ascorbic acid). The experiments were repeated on, at least, three batches of PEC-NGs.

## 3. Results and Discussion

In the present work, the physicochemical characteristics of PEC-NGs after vs. before the lyophilization and reconstitution process were compared to evaluate the efficiency in preserving their colloidal structures. Firstly, HA/DEAE-D PEC-NGs were studied to highlight the effects of basic parameters, such as the polyanion/polycation ratio in PECs (represented by the molar charge ratio n−/n+) and cryoprotectant type as well as cryoprotectant concentration. The optimal conditions observed were then reinvestigated on different PEC-NGs with PLL replacing DEAE-D and/or the thermoresponsive HA-M2005 replacing HA since HA-M2005/PLL constituted the most potent system for drug delivery from our previous work [4]. This revealed the effects of polycation type and the presence of M2005 grafts on the stability of PEC-NGs after freeze-drying. At this point, the impact of C_P_ before freeze-drying was studied to determine its optimal value for the possibility of concentrating CUR-loaded PEC-NGs after lyophilization. Finally, CUR release kinetics in vitro before and after lyophilization was also evaluated.

### 3.1. Effect of Charge Ratio

Lyophilization was realized for the suspensions of HA/DEAE-D PEC-NGs having different n−/n+ ratios (1.25, 2.5, and 5) at C_P_ = 0.5 g.L^−1^ without cryoprotectant. These n−/n+ values higher than 1 were chosen for this study since they were previously reported to result in stable PEC-NGs containing mostly HA, which is our main polymer of interest [4,15]. However, after lyophilization, only PEC-NGs with n−/n+ = 5 can be redispersed in Milli-Q water to form homogeneously translucent suspensions, while visible large aggregates remained after redispersing the freeze-dried PEC-NGs having n−/n+ of 1.25 or 2.5. The experiment was then reproduced on these two types of PEC-NG in the presence of trehalose as a cryoprotectant at different concentrations, increasing with a 1% (m/v) interval until obtaining a visually homogeneous reconstitution. The characteristics of rehydrated PEC-NGs at the optimal trehalose concentrations as well as their change factor R (ratio of values after reconstitution to those before freeze-drying) are reported in Table 1. Trehalose was preliminarily chosen as the cryoprotectant since it is generally the most effective among cryoprotectants according to the literature [11,16,17]. Further studies regarding cryoprotectant selection are described in Section 3.2.

It can be seen from Table 1 that PEC-NGs with a higher n−/n+ would require less trehalose during lyophilization for homogeneous redispersion in water during the reconstitution, which was confirmed by particle sizes remaining in the colloidal range with relatively low PDIs. This can be explained by the better electrostatic stabilization of these PEC-NGs since an n−/n+ further from 1 would lead to greater surface net charges, namely, zeta potential changed from −30 to −45 mV when n−/n+ is increased from 1.25 to 5 as reported in our earlier work [15]. Indeed, during the freezing of PEC-NG suspensions, the continuous formation of pure ice crystals should lead to a gradual reduction in the volume of the liquid phase, thus, a local increase in particle and counter-ion concentrations, also known as cryoconcentration [10]. This will result in different stresses on PEC-NGs: (i) mechanical damage or deformation of PEC-NGs caused by growing ice crystals, (ii) structure interference caused by the loss of hydrogen bonds as water molecules are transferred to the ice phase and then sublimated, and (iii) closer approach of particles and weakened electrostatic stabilization by the charge screening effect of counter-ions which facilitate particle aggregation [10,18]. Logically, a higher absolute zeta potential at the beginning would allow a greater electrostatic repulsion to avoid such aggregation and also facilitate disaggregation during the reconstitution, which was also observed by Umerska et al. [11] and Eliyahu et al. [19]. Furthermore, the higher proportion of HA on the NG surface may also contribute to their better stability during freezing since HA can also have cryoprotective effects [1], e.g., by maintaining intra- and inter-molecular hydrogen bonds on the particle surface during dehydration [20]. However, after freeze-drying and reconstitution, we still observed an increase of nearly 40% in the particle size of HA/DEAE-D PEC-NGs at n−/n+ = 2.5 (Table 1), which was also the most interesting n−/n+ for CUR encapsulation reported in our earlier studies [4]. Therefore, different cryoprotectant types and concentrations were further tested, particularly on PEC-NGs at this n−/n+ in an attempt to better preserve their particle size (Section 3.2).

### 3.2. Effects of Cryoprotectant Type and Concentration

HA/DEAE-D PEC-NGs at n−/n+ = 2.5 and C_P_ = 0.5 g.L^−1^ were freeze-dried with 1% m/v of different cryoprotectants, i.e., trehalose, sucrose, glucose, and PEGs with M_n_ of 2000; 10,000 or 20,000 g.mol^−1^, which have been largely studied as cryoprotectants for nanoformulations in the literature [21,22]. In our case, the addition of PEG caused visible aggregation of PEC-NGs after reconstitution (PEG of M_n_ = 20,000 g.mol^−1^) or even before freeze-drying (PEGs of M_n_ of 2000 or 10,000 g.mol^−1^). On the contrary, trehalose, sucrose, and glucose did not cause visible aggregation or changes in the particle size before lyophilization (Table 2).

**Figure 2:**
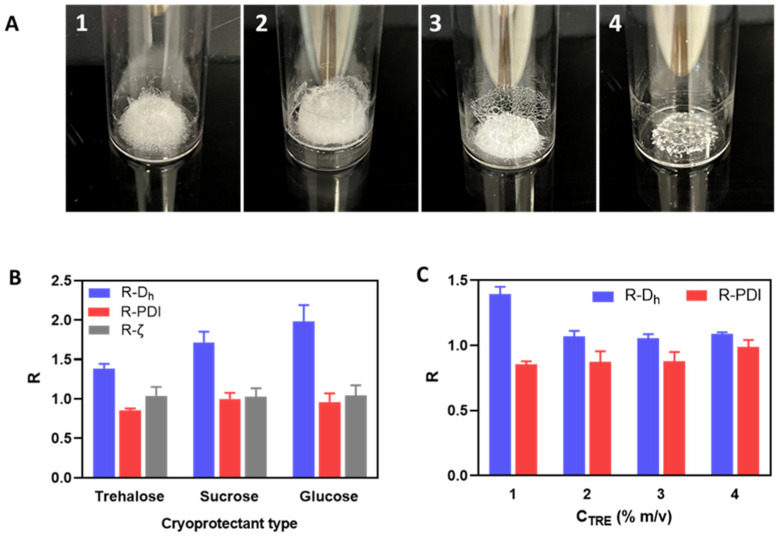
(**A**): Freeze-dried cakes of HA/DEAE-D PEC-NGs at C_P_ = 0.5 g.L^−1^ and n−/n+ = 2.5 without cryoprotectant (**A1**), or with 1% m/v trehalose (**A2**), sucrose (**A3**), or glucose (**A4**). (**B**,**C**): Change factor R (value after reconstitution: before freeze-drying) of PEC-NG characteristics when freeze-dried with different types of cryoprotectant at 1% m/v (**B**) or with trehalose at different concentrations C_TRE_ (**C**) (*n* ≥ 3). D_h_: hydrodynamic diameter, PDI: polydispersity index, ζ: zeta potential.

**Figure 3:**
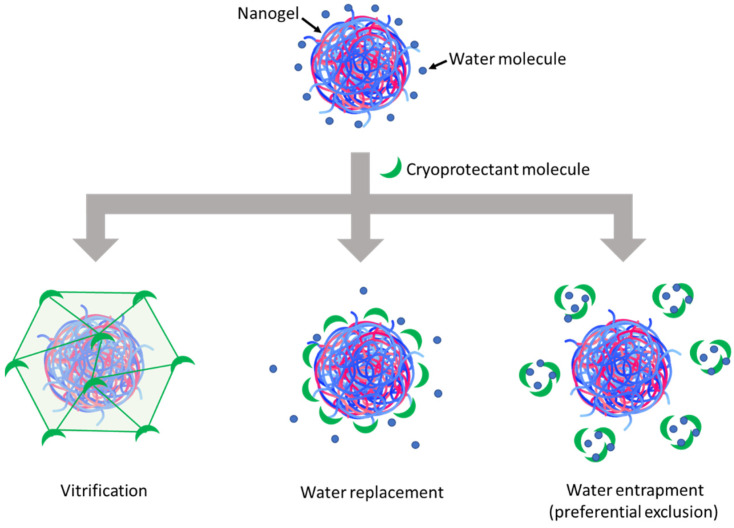
Schematic representation of cryoprotection mechanisms of cryoprotectants.

The resulting lyophilized cakes (Figure 2A) could be redispersed to establish visually homogeneous suspensions, which were then characterized by DLS to evaluate the change factors of particle size and zeta potential after lyophilization and reconstitution (Figure 2B). Regarding the visual aspects of freeze-dried cakes (Figure 2A), using disaccharide cryoprotectants (i.e., sucrose and trehalose) also resulted in porous cakes as the one obtained without cryoprotectant, while using glucose led to a collapsed cake. After reconstitution, although the PDI and zeta potential were well-maintained, a significant increase in particle size was always observed with all three cryoprotectants (Figure 2B). However, it can still be seen that trehalose was the most effective among these cryoprotectants in preserving the particle size of HA/DEAE-D PEC-NGs while glucose was the least effective, which is in accordance with the visual aspect of lyophilized cakes earlier. Then, by increasing the trehalose concentration, we saw that the particle size can be well-preserved (change factor in the range of 1–1.1) with at least 2% m/v trehalose (Figure 2C). To explain the cryoprotective mechanisms of these cryoprotectants, three theories have been described in the literature: vitrification, water replacement, and water entrapment or preferential exclusion (Figure 3) [23,24].

In the vitrification theory, during cryoconcentration, the highly increased concentration of cryoprotectants can render the liquid phase more viscous and then form a glassy layer around the NGs when water is completely removed. This can reduce the mobility of particles in the liquid phase to avoid their close approach, thus preventing their aggregation, and eventually constitutes a solid glassy matrix supporting the whole system and stabilizing the NGs in a dry state [7]. In this theory, the glass transition temperature (T_g_) of cryoprotectants is an important parameter since a higher T_g_ would lead to a higher stability of the glassy matrix to better preserve the products sequestered inside. Disaccharides are, thus, more preferable cryoprotectants compared with monosaccharides since the former normally have a higher T_g_ [10]. Concerning the three saccharide cryoprotectants used in this work, the order of their efficacy in preserving NG particle size as shown in Figure 2B is consistent with their T_g_ found in the literature: T_g_ of glucose (30 °C) < T_g_ of sucrose (58 °C) < T_g_ of trehalose (108 °C) [25]. The low T_g_ of glucose can also lead to a low T_g_’ (apparent T_g_ of concentrated cryoprotectants with NGs and other solutes), which may explain the collapse of the lyophilized cake in this case [10]. Although cake collapse is only an aesthetic defect of lyophilized products, which usually has no significant impact on their physicochemical stability, it can, however, lead to a longer time needed for the drying as well as the redispersion steps due to low porosity [26,27]. In the water replacement mechanism, saccharide molecules can replace water to maintain the hydrogen bonds on NG surfaces, thus keeping the NGs in a “pseudo-hydrated” state to avoid interference with their structure upon dehydration as well as reduce particle aggregation through hydrophobic interactions [28,29]. Meanwhile, the water entrapment or preferential exclusion theory has been less widely described and is usually applied for protein cryoprotection [24]. According to this theory, sugar molecules like trehalose can self-organize to “entrap” water molecules, thus reducing the hydration rate of the protein molecules and favoring their folding in a more compact and rigid state, which is more stable and can protect them from the damage caused by ice crystals [30]. This may also be the case for the cryoprotection of our NGs. In theories of water replacement and water entrapment, in order to explain the different effects between sucrose and trehalose despite their similar chemical formula, some studies have suggested that the geometrical structure of sucrose, which is different from that of trehalose, may allow for intramolecular hydrogen bonds that limit its capacity to form hydrogen bonds with water as well as other macromolecules and, therefore, results in lower cryoprotection efficiency [31,32].

Regarding PEG, despite its cryoprotectant effect reported for some systems, its presence might also favor agglomeration or aggregation of nanoformulations [22]. Indeed, it has been known that PEG is incompatible with dextran since the presence of both these polymers in the solution can cause phase separation, known as aqueous two-phase systems [10,33], which might explain the formation of large aggregates observed upon adding PEG in HA/DEAE-D PEC-NG suspensions. PEG has also been reported to act as a crowding agent to intensify the complexation between HA and PLL and, hence, favor phase separation [34]. Furthermore, PEG may also bridge nanoparticles to cause flocculation, known as polymer bridging flocculation [7,35]. In the case of PEG having M_n_ of 20,000 g.mol^−1^, the resulting high viscosity may also be a factor preventing homogeneous redispersion of lyophilized PEC-NGs.

From the above results, it can be generally seen that PEG is less effective than the three sugars tested for the cryoprotection of PEC-NGs, while trehalose seems to be the most appropriate cryoprotectant. The noticeable cryoprotective effect of trehalose has also been reported for other HA-based systems, such as HA/CTS nanoparticles [22], cisplatin-conjugated HA nanoparticles [36], or micellar NGs from HA [37]. In regards to cryoprotectant concentration, according to the cryoprotecting mechanisms, cryoprotection efficiency should be optimal only at a sufficiently high cryoprotectant concentration, which is around 2% (m/v) trehalose for HA/DEAE-D PEC-NGs in our case (Figure 2C). From these results, trehalose at concentrations (C_TRE_) between 0–2% m/v was selected for later studies.

### 3.3. Effects of M2005 and Polycation Type

**Figure 4:**
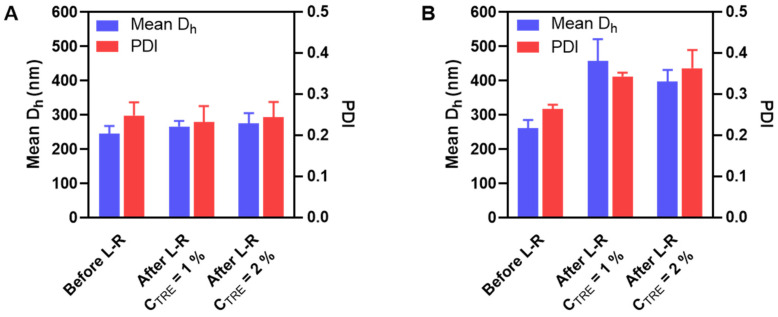
Mean D_h_ and PDI before and after lyophilization and reconstitution (L-R) of HA-M2005/DEAE-D PEC-NGs (**A**) and HA-M2005/PLL PEC-NGs (**B**) at n−/n+ = 2.5 and C_P_ = 0.5 g.L^−1^ with different trehalose concentrations (*n* ≥ 3). The results of freshly prepared PEC-NGs (before L-R) were retrieved from our previous work as references [4].

HA-M2005/DEAE-D and HA-M2005/PLL PEC-NGs (C_P_ = 0.5 g.L^−1^, n−/n+ = 2.5) with C_TRE_ of 0, 1 or 2% m/v were freeze-dried and reconstituted. During their reconstitution, like HA/DEAE-D PEC-NGs in the previous sections, homogeneous redispersion could not be obtained without trehalose but only with C_TRE_ of at least 1%, thus allowing for characterization by DLS (Figure 4).

For HA-M2005/DEAE-D PEC-NGs, C_TRE_ = 1% was sufficient to keep their particle size unchanged (Figure 4A), which means a more efficient preservation compared with HA/DEAE-D PEC-NGs in Section 3.2, which required at least 2% trehalose. Such a more positive result for HA-M2005/DEAE-D PEC-NGs is probably due to the stabilizing effect of the M2005 grafts through their hydrophobic interactions and LCST behaviors [4]. However, for HA-M2005/PLL PEC-NGs, even a trehalose concentration as high as 2% could not prevent a considerable increase (of around 50%) in particle size (Figure 4B), which may be attributed to the highly hydrophobic nature of PLL in HA-M2005/PLL PECs [4] which facilitates their aggregation under freezing stress. We, therefore, tried a higher C_TRE_ (i.e., 4% m/v) but such a high trehalose concentration seemed to destabilize the PEC-NGs, as this led to the visible aggregation of HA-M2005/DEAE-D PEC-NGs and a significant increase in the particle size of HA-M2005/PLL PEC-NGs (from 262 ± 24 to 380 ± 29 nm). It has been described in the literature that exceeding the optimal cryoprotectant concentration can sometimes destabilize nanoformulations, whose mechanisms have not always been well clarified [7,38,39]. With M2005 grafts, which constitute the amphiphilic and thermoresponsive properties of PEC-NGs, the aforementioned water entrapment effect of trehalose may be comparable to the salting-out effect of counterions, which over-favors hydrophobic interactions and, thus, aggregation. Moreover, in the case of HA-M2005/PLL PEC-NGs at C_P_ = 0.5 g.L^−1^ and n−/n+ = 2.5, also the most interesting system among different PEC-NGs studied in our previous work [4], C_TRE_ = 1–2% seems to be the most suitable concentration for their freeze-drying, although the efficiency in preserving particle size was not completely optimal. In addition, with those reconstituted HA-M2005/PLL PEC-NGs, we reverified the NG shrinkage upon heating at 50 °C (Figure S1), which is typical for their thermoresponsive behaviors [4]. This confirmed that the thermoresponsiveness of HA-M2005-based PEC-NGs still remained after lyophilization.

### 3.4. Reduction of Polymer Concentration before Freeze-Drying

**Figure 5:**
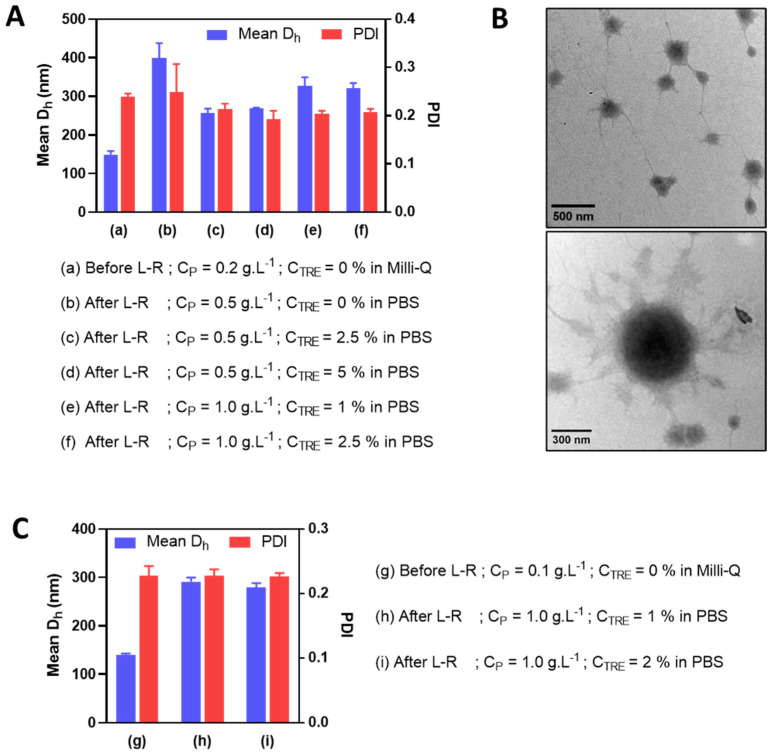
Mean D_h_ and PDI before and after lyophilization and reconstitution (L-R) in PBS at different C_P_ and C_TRE_ of HA-M2005/PLL PEC-NGs (n−/n+ = 2.5) initially prepared at C_P_ = 0.2 g.L^−1^ (**A**) or 0.1 g.L^−1^ (**C**) (*n* ≥ 3). The morphologies of the reconstituted PEC-NGs at C_P_ = 1 g.L^−1^ and C_TRE_ = 1% from initial C_P_ = 0.2 g.L^−1^ were verified by TEM (**B**).

In an attempt to further reduce the particle size (i.e., to under 350 nm with PDI under 0.3) of HA-M2005/PLL PEC-NGs at n−/n+ = 2.5 after freeze-drying and reconstitution, PEC-NGs were prepared at a lower polymer concentration (i.e., C_P_ of 0.2 g.L^−1^). This was realized upon the presumption that such a lower C_P_ would result in a smaller particle size before lyophilization and also after reconstitution, which may allow for further concentrating PEC-NGs without causing large aggregates. This time, we also tried using PBS as a dispersion medium during PEC-NG reconstitution for their future applications, such as biological studies in vitro or in vivo. As a first attempt, the PBS volume added for reconstitution was equal to 40% of the suspension volume before lyophilization, thus, a final C_P_ of 0.5 g.L^−1^. In this case, PEC-NGs could be homogeneously redispersed even without trehalose but still showed a relatively large particle size (around 400 nm), which means a 2.5-fold increase with respect to the initial size of PEC-NGs at C_P_ = 0.2 g.L^−1^ before freeze-drying (Figure 5A). Otherwise, the presence of trehalose at 1 or 2% m/v before lyophilization (i.e., C_TRE_ of 2.5 or 5% after reconstitution) resulted in less increased particle size (around 250 nm) (Figure 5A) with good stability for, at least, 7 days (Figure S2). Therefore, in comparison with PEC-NGs freeze-dried and reconstituted at C_P_ = 0.5 g.L^−1^ in Section 3.3, PEC-NGs reconstituted after lyophilizing more diluted suspensions (C_P_ = 0.2 g.L^−1^) could show a lower final particle size despite the same final C_P_ during reconstitution.

After such an observation, we tried further concentrating PEC-NGs by reducing PBS volume during reconstitution to 20% of the initial volume before freeze-drying to reach a final C_P_ of 1 g.L^−1^, in the presence of trehalose with final C_TRE_ of 1 or 2.5% m/v. Such a reduction in reconstitution volume seems to slightly increase the final particle size of PEC-NGs (i.e., to around 300 nm) (Figure 5A), which remained acceptable. The morphologies of PEC-NGs reconstituted at C_P_ = 1 g.L^−1^ were also verified by TEM (Figure 5B). It was also noticed that PDI was always relatively stable around 0.2, indicating good uniformity of particle size regardless of the studied C_P_ and C_TRE_. We also tried further reducing the initial C_P_ to 0.1 g.L^−1^ but this did not lead to a considerable difference regarding particle size and PDI, which were still around 300 nm and 0.2, respectively (Figure 5C). In general, these results show that the approach of lyophilization of diluted PEC-NG suspensions can be interesting for concentrating PEC-NGs and, probably, the encapsulated drugs.

### 3.5. Curcumin Encapsulation and Release

**Figure 6:**
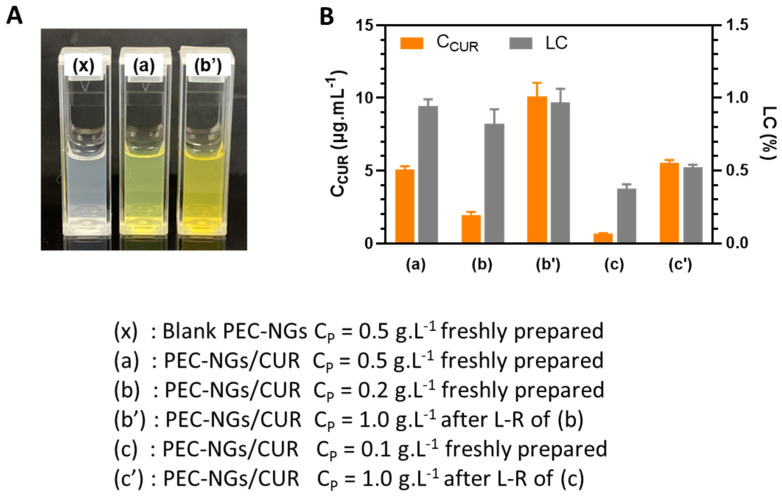
Blank or CUR-loaded HA-M2005/PLL PEC-NGs (n−/n+ = 2.5) before and after lyophilization and reconstitution (L-R) in PBS: (**A**) Visual aspect and (**B**) CUR concentration (C_CUR_) and loading capacity (LC) (*n* ≥ 3). Samples at C_P_ = 0.5 g.L^−1^ were retrieved from our previous work [4] as references.

In order to verify the possibility of concentrating encapsulated drugs in PEC-NGs after lyophilization, CUR-loaded HA-M2005/PLL PEC-NGs (n−/n+ = 2.5) were prepared at C_P_ of 0.1 or 0.2 g.L^−1^, then freeze-dried with C_TRE_ of 0.1 or 0.2% m/v, respectively, and reconstituted with PBS to reach a final C_P_ of 1 g.L^−1^ and C_TRE_ of 1% as for blank PEC-NGs in the previous section. As can be seen from Figure 6A, compared with suspensions of the same PEC-NG type freshly prepared at C_P_ = 0.5 g.L^−1^, the suspension reconstituted at C_P_ = 1 g.L^−1^ from initial C_P_ = 0.2 g.L^−1^ was also visually homogeneous but with a darker yellow color, suggesting qualitatively an effective increase in CUR concentration without causing precipitation. After CUR quantification, the results obtained with the suspensions both before lyophilization and after reconstitution from initial C_P_ = 0.2 g.L^−1^ were also consistent with those obtained from PEC-NGs freshly prepared at C_P_ = 0.5 g.L^−1^ (Figure 6B), since LC values were in the same range and the CUR content was, thus, always in good proportion with C_P_. However, PEC-NGs prepared at C_P_ = 0.1 g.L^−1^ showed a much lower LC and, therefore, resulted in suspensions with a lower CUR concentration after reconstitution even at the same final C_P_ of 1 g.L^−1^ (Figure 6B). The low loading capacity of PEC-NGs at C_P_ = 0.1 g.L^−1^ may stem from their more porous and looser structure compared with those prepared at C_P_ = 0.2 g.L^−1^, suggested by their similar particle sizes (Figure 5A, C) despite the much lower C_P_ in the former case. From such results, freeze-drying suspensions at C_P_ = 0.2 g.L^−1^ with 0.2% trehalose can be considered the most appropriate method for lyophilizing HA-M2005/PLL PEC-NGs of n−/n+ = 2.5, which can preserve satisfactorily both their particle size and CUR content.

**Figure 7:**
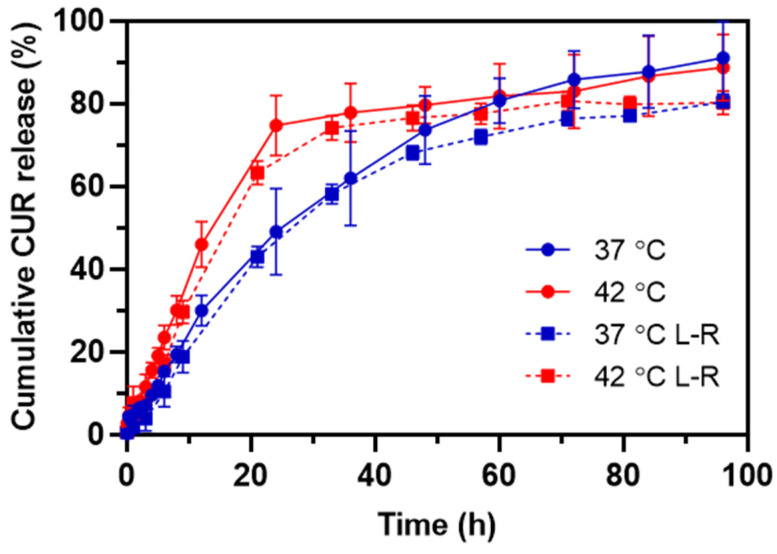
The release profile of CUR at 37 °C and 42 °C from HA-M2005/PLL PEC-NGs (n−/n+ = 2.5) freshly prepared at C_P_ = 0.5 g.L^−1^ (solid lines) or after lyophilization and reconstitution (L-R) at C_P_ = 1 g.L^−1^ from initial C_P_ = 0.2 g.L^−1^ (dashed lines) (*n* ≥ 3).

To verify the thermo-triggered release of CUR from HA-M2005/PLL PEC-NGs (n−/n+ = 2.5) as well as the change in such behavior after lyophilization, the release profiles of CUR at 37 °C and 42 °C, i.e., maximum temperature for in vivo local hyperthermia [3,40], were characterized for PEC-NGs freshly prepared at C_P_ = 0.5 g.L^−1^ and those reconstituted at C_P_ = 1 g.L^−1^ in PBS after lyophilization at C_P_ = 0.2 g.L^−1^ with 0.2% trehalose. The cumulative CUR release over time is presented in Figure 7.

In the case of PEC-NGs at C_P_ = 0.5 g.L^−1^ without lyophilization, the release kinetics at 37 °C was relatively fast at the beginning and gradually slower over time, resulting in 90% of the encapsulated CUR being released after 4 days. As these PEC-NGs have a core-shell structure with a dense and hydrophobic core inside a corona-like hydrophilic shell [4], the first rapid phase of release can be attributed to the diffusion of CUR molecules adsorbed on the hydrophilic shell of NGs while the slow release afterward was probably because of CUR molecules entrapped in NG cores, which have a great affinity to CUR through hydrophobic interactions and, thus, decelerated its diffusion. Meanwhile, the release at 42 °C showed more evident biphasic kinetics with the first phase being significantly more rapid than that at 37 °C. The faster release of CUR at 42 °C is in agreement with the thermoresponsive behaviors of these PEC-NGs, as NG shrinkage, when the temperature is increased [4], may cause the expulsion of water as well as CUR molecules from the NG interior. This is in accordance with a recent work by Khodaei et al. on CUR release from thermoresponsive nanoparticles from PEO-PPO-PEO copolymers, which showed that the CUR release rate was increased upon a temperature rise to 45 °C, but started to decrease when the temperature was higher than 45 °C [41]. Through mathematical modeling, the authors suggested that the former acceleration of CUR release is related to particle shrinkage, while the latter deceleration is due to stronger hydrophobic interactions between CUR and the copolymers. The higher release rate of drugs upon temperature increase has also been observed with other thermoresponsive systems from LCST-type polymers, e.g., methotrexate release from polymeric micelles of poly(N-isopropylacrylamide-*co*-acrylamide)-*b*-poly(n-butylmethacrylate) [40] or release of CUR and doxorubicin from poly(N-isopropylacrylamide)-coated particles [42]. The results in the present work suggest thus the interest of HA-M2005/PLL PEC-NGs for thermo-triggered drug release, which can be applied for spatiotemporally controlling drug release by applying local hyperthermia in vivo [3]. Compared with those results, the release of CUR was not significantly different when PEC-NGs were concentrated at C_P_ = 1 g.L^−1^ after lyophilization (Figure 7), which shows that the suggested method for freeze-drying and concentrating PEC-NGs upon reconstitution has no considerable effect on drug release as well as its thermoresponsive characteristics. The smaller standard deviations, in this case, were probably due to the higher CUR concentration as the samples were concentrated, allowing for a more precise absorbance measurement.

Concerning the CUR release mechanism from NGs, this can be governed by a complex set of factors, namely simple diffusion, NG swelling, degradation, or disassembly, as well as external stimuli like temperature in the case of thermoresponsive NGs [43]. In order to better predict the release mechanism from the present PEC-NGs, both the release curves at 37 and 42 °C of PEC-NGs of C_P_ = 0.5 g.L^−1^ were fitted to different mathematical models most widely used for describing drug release, including a zero-order model, a first-order model, a Higuchi model, and a Korsmeyer-Peppas model (Figure S3) [44]. It was revealed that CUR release at 37 °C follows first-order kinetics, which confirmed that the release rate was not constant but depended on the actual CUR content remaining in the NGs [43,44]. However, none of the models used could fit the release data at 42 °C, probably due to the copresence of two strictly distinct phases in this case which may correspond to two completely different mechanisms. Therefore, more complete modeling studies should be carried out if the release mechanism at 42 °C needs to be better understood.

## 4. Conclusions

This work concerns the effects of different formulation factors on the preservation of HA-based PEC-NGs after lyophilization and reconstitution, especially thermoresponsive PEC-NGs from HA-M2005 and PLL. It was revealed that the most practical approach for the lyophilization of such systems is freeze-drying PEC-NG suspensions prepared in a dilute regime (C_P_ = 0.2 g.L^−1^) with 0.2% m/v trehalose as a cryoprotectant. This approach allowed for concentrating PEC-NGs to at least C_P_ = 1 g.L^−1^ in PBS and, thus, increasing the drug content (i.e., curcumin) in the reconstitution step for future biological studies, while maintaining the particle size in an acceptable range (i.e., under 350 nm). Moreover, such a process of freeze-drying and concentrating PEC-NGs upon reconstitution did not considerably change the drug release profile as well as its thermoresponsiveness, which would be an interesting behavior for their applications in controlled drug delivery. However, further studies need to be performed in order to predict the biorelevance of the reconstituted suspensions before biological studies, namely, osmolality evaluation since the osmolality after concentrating PEC-NGs and cryoprotectants might exceed the physiological limits. In addition, before biological studies, it would be highly relevant to find a suitable technique for sterilizing these systems, e.g., by autoclave since it has been shown to be efficient for both stabilizing and sterilizing NGs from amphiphilic HA derivatives [45,46]. Moreover, for better preserving PEC-NG structure, it would also be interesting to further investigate the technical aspect of the lyophilization process, e.g., the effects of freezing speed, drying time, temperature, pressure, and secondary drying phase (moisture desorption) on the microstructures of these systems.

## Figures and Tables

**Table 1:** Characteristics of HA/DEAE-D PEC-NGs (C_P_ = 0.5 g.L^−1^) reconstituted after freeze-drying at the lowest trehalose concentrations allowing homogeneous reconstitution (*n* ≥ 3).

n−/n+	C*_TRE_ (% m/v)	Mean D_h_ (nm)	R-D_h_	PDI	R-PDI
1.25	8	182 ± 6	1.07 ± 0.02	0.09 ± 0.01	0.84 ± 0.12
2.5	1	315 ± 15	1.39 ± 0.06	0.21 ± 0.01	0.85 ± 0.03
5	0	264 ± 37	1.04 ± 0.11	0.30 ± 0.01	0.94 ± 0.06

C*_TRE_: lowest trehalose concentration for homogeneous redispersion of freeze-dried PEC-NGs. R-D_h_: change factor of D_h_ after lyophilization and reconstitution. R-PDI: change factor of PDI after lyophilization and reconstitution.

**Table 2:** Change factors (R_i_) of mean D_h_ and PDI upon adding 1% m/v cryoprotectant (*n* ≥ 3).

Cryoprotectant	R_i_-D_h_	R_i_-PDI
Trehalose	1.01 ± 0.02	0.97 ± 0.04
Sucrose	0.98 ± 0.02	1.02 ± 0.02
Glucose	1.00 ± 0.02	0.99 ± 0.04

## Data Availability

Not applicable.

## Supplementary Materials

The following supporting information can be downloaded at: https://www.mdpi.com/article/10.3390/pharmaceutics15030929/s1, Figure S1: Thermoresponsiveness of NG particle size after lyophilization and reconstitution; Figure S2: Stability of NG after lyophilization and reconstitution; Figure S3: Model fitting of curcumin release data.

## References

[1] Le H.V., Le Cerf D. (2022). Colloidal Polyelectrolyte Complexes from Hyaluronic Acid: Preparation and Biomedical Applications. Small.

[2] Karimi M., Sahandi Zangabad P., Ghasemi A., Amiri M., Bahrami M., Malekzad H., Ghahramanzadeh Asl H., Mahdieh Z., Bozorgomid M., Ghasemi A. (2016). Temperature-responsive smart nanocarriers for delivery of therapeutic agents: Applications and recent advances. ACS Appl. Mater. Interfaces.

[3] Bordat A., Boissenot T., Nicolas J., Tsapis N. (2019). Thermoresponsive polymer nanocarriers for biomedical applications. Adv. Drug Deliv. Rev..

[4] Le H.V., Dulong V., Picton L., Le Cerf D. (2022). Thermoresponsive nanogels based on polyelectrolyte complexes between polycations and functionalized hyaluronic acid. Carbohydr. Polym..

[5] Wickens J.M., Alsaab H.O., Kesharwani P., Bhise K., Amin M.C.I.M., Tekade R.K., Gupta U., Iyer A.K. (2017). Recent advances in hyaluronic acid-decorated nanocarriers for targeted cancer therapy. Drug Discov. Today.

[6] Simulescu V., Kalina M., Mondek J., Pekař M. (2016). Long-term degradation study of hyaluronic acid in aqueous solutions without protection against microorganisms. Carbohydr. Polym..

[7] Abdelwahed W., Degobert G., Stainmesse S., Fessi H. (2006). Freeze-drying of nanoparticles: Formulation, process and storage considerations. Adv. Drug Deliv. Rev..

[8] Veilleux D., Nelea M., Biniecki K., Lavertu M., Buschmann M.D. (2016). Preparation of concentrated chitosan/DNA nanoparticle formulations by lyophilization for gene delivery at clinically relevant dosages. J. Pharm. Sci..

[9] Bhatnagar B.S., Bogner R.H., Pikal M.J. (2007). Protein stability during freezing: Separation of stresses and mechanisms of protein stabilization. Pharm. Dev. Technol..

[10] Trenkenschuh E., Friess W. (2021). Freeze-drying of nanoparticles: How to overcome colloidal instability by formulation and process optimization. Eur. J. Pharm. Biopharm..

[11] Umerska A., Paluch K.J., Santos-Martinez M.J., Corrigan O.I., Medina C., Tajber L. (2018). Freeze drying of polyelectrolyte complex nanoparticles: Effect of nanoparticle composition and cryoprotectant selection. Int. J. Pharm..

[12] Gheran C.V., Voicu S.N., Galateanu B., Callewaert M., Moreau J., Cadiou C., Chuburu F., Dinischiotu A. (2022). In Vitro Studies Regarding the Safety of Chitosan and Hyaluronic Acid-Based Nanohydrogels Containing Contrast Agents for Magnetic Resonance Imaging. Int. J. Mol. Sci..

[13] Puiggali-Jou A., Micheletti P., Estrany F., Del Valle L.J., Aleman C. (2017). Electrostimulated release of neutral drugs from Polythiophene nanoparticles: Smart regulation of drug–polymer interactions. Adv. Healthc. Mater..

[14] Rabanel J.-M., Faivre J., Paka G.D., Ramassamy C., Hildgen P., Banquy X. (2015). Effect of polymer architecture on curcumin encapsulation and release from PEGylated polymer nanoparticles: Toward a drug delivery nano-platform to the CNS. Eur. J. Pharm. Biopharm..

[15] Le H.V., Dulong V., Picton L., Le Cerf D. (2021). Polyelectrolyte complexes of hyaluronic acid and diethylaminoethyl dextran: Formation, stability and hydrophobicity. Colloids Surf. A.

[16] Merivaara A., Zini J., Koivunotko E., Valkonen S., Korhonen O., Fernandes F.M., Yliperttula M. (2021). Preservation of biomaterials and cells by freeze-drying: Change of paradigm. J. Control. Release.

[17] Mudassir J., Darwis Y., Muhamad S., Khan A.A. (2019). Self-assembled insulin and nanogels polyelectrolyte complex (Ins/NGs-PEC) for oral insulin delivery: Characterization, lyophilization and in-vivo evaluation. Int. J. Nanomed..

[18] Picco A.S., Ferreira L.F., Liberato M.S., Mondo G.B., Cardoso M.B. (2018). Freeze-drying of silica nanoparticles: Redispersibility toward nanomedicine applications. Nanomedicine.

[19] Eliyahu S., Almeida A., Macedo M.H., das Neves J., Sarmento B., Bianco-Peled H. (2020). The effect of freeze-drying on mucoadhesion and transport of acrylated chitosan nanoparticles. Int. J. Pharm..

[20] Peer D., Florentin A., Margalit R. (2003). Hyaluronan is a key component in cryoprotection and formulation of targeted unilamellar liposomes. Biochim. Biophys. Acta Biomembr..

[21] Luo W.-C., Beringhs A.O.R., Kim R., Zhang W., Patel S.M., Bogner R.H., Lu X. (2021). Impact of formulation on the quality and stability of freeze-dried nanoparticles. Eur. J. Pharm. Biopharm..

[22] Almalik A., Alradwan I., Kalam M.A., Alshamsan A. (2017). Effect of cryoprotection on particle size stability and preservation of chitosan nanoparticles with and without hyaluronate or alginate coating. Saudi Pharm. J..

[23] Jain N.K., Roy I. (2009). Effect of trehalose on protein structure. Protein Sci..

[24] Vinciguerra D., Gelb M.B., Maynard H.D. (2022). Synthesis and Application of Trehalose Materials. JACS Au.

[25] Zhang M., Oldenhof H., Sydykov B., Bigalk J., Sieme H., Wolkers W.F. (2017). Freeze-drying of mammalian cells using trehalose: Preservation of DNA integrity. Sci. Rep..

[26] Tejedor M.B., Fransson J., Millqvist-Fureby A. (2020). Freeze-dried cake structural and physical heterogeneity in relation to freeze-drying cycle parameters. Int. J. Pharm..

[27] Schersch K., Betz O., Garidel P., Muehlau S., Bassarab S., Winter G. (2013). Systematic investigation of the effect of lyophilizate collapse on pharmaceutically relevant proteins III: Collapse during storage at elevated temperatures. Eur. J. Pharm. Biopharm..

[28] Rampino A., Borgogna M., Blasi P., Bellich B., Cesàro A. (2013). Chitosan nanoparticles: Preparation, size evolution and stability. Int. J. Pharm..

[29] Crowe J.H., Clegg J.S., Crowe L.M., Reid D. (1998). Anhydrobiosis: The water replacement hypothesis. The Properties of Water in Foods ISOPOW 6.

[30] Chiu P.-L., Kelly D.F., Walz T. (2011). The use of trehalose in the preparation of specimens for molecular electron microscopy. Micron.

[31] Mathlouthi M. (1981). X-ray diffraction study of the molecular association in aqueous solutions of d-fructose, d-glucose, and sucrose. Carbohydr. Res..

[32] Lerbret A., Bordat P., Affouard F., Descamps M., Migliardo F. (2005). How homogeneous are the trehalose, maltose, and sucrose water solutions? An insight from molecular dynamics simulations. J. Phys. Chem. B.

[33] Diamond A., Hsu J. (1989). Phase diagrams for dextran-PEG aqueous two-phase systems at 22 °C. Biotechnol. Tech..

[34] Park S., Barnes R., Lin Y., Jeon B.-J., Najafi S., Delaney K.T., Fredrickson G.H., Shea J.-E., Hwang D.S., Han S. (2020). Dehydration entropy drives liquid-liquid phase separation by molecular crowding. Commun. Chem..

[35] Smalley M., Hatharasinghe H., Osborne I., Swenson J., King S. (2001). Bridging Flocculation in Vermiculite-PEO Mixtures. Langmuir.

[36] Ishiguro S., Cai S., Uppalapati D., Turner K., Zhang T., Forrest W.C., Forrest M.L., Tamura M. (2016). Intratracheal administration of hyaluronan-cisplatin conjugate nanoparticles significantly attenuates lung cancer growth in mice. Pharm. Res..

[37] Grimaudo M.A., Amato G., Carbone C., Diaz-Rodriguez P., Musumeci T., Concheiro A., Alvarez-Lorenzo C., Puglisi G. (2020). Micelle-nanogel platform for ferulic acid ocular delivery. Int. J. Pharm..

[38] Mohammady M., Yousefi G. (2020). Freeze-drying of pharmaceutical and nutraceutical nanoparticles: The effects of formulation and technique parameters on nanoparticles characteristics. J. Pharm. Sci..

[39] Lee M.K., Kim M.Y., Kim S., Lee J. (2009). Cryoprotectants for freeze drying of drug nano-suspensions: Effect of freezing rate. J. Pharm. Sci..

[40] Sun F., Wang Y., Wei Y., Cheng G., Ma G. (2014). Thermo-triggered drug delivery from polymeric micelles of poly (N-isopropylacrylamide-co-acrylamide)-b-poly (n-butyl methacrylate) for tumor targeting. J. Bioact. Compat. Polym..

[41] Khodaei A., Jahanmard F., Hosseini H.M., Bagheri R., Dabbagh A., Weinans H., Yavari S.A. (2022). Controlled temperature-mediated curcumin release from magneto-thermal nanocarriers to kill bone tumors. Bioact. Mater..

[42] Asghar K., Qasim M., Dharmapuri G., Das D. (2017). Investigation on a smart nanocarrier with a mesoporous magnetic core and thermo-responsive shell for co-delivery of doxorubicin and curcumin: A new approach towards combination therapy of cancer. RSC Adv..

[43] Lee J.H., Yeo Y. (2015). Controlled drug release from pharmaceutical nanocarriers. Chem. Eng. Sci..

[44] Bruschi M.L. (2015). 5—Mathematical models of drug release. Strategies to Modify the Drug Release from Pharmaceutical Systems.

[45] Montanari E., De Rugeriis M.C., Di Meo C., Censi R., Coviello T., Alhaique F., Matricardi P. (2015). One-step formation and sterilization of gellan and hyaluronan nanohydrogels using autoclave. J. Mater. Sci. Mater. Med..

[46] Zoratto N., Forcina L., Matassa R., Mosca L., Familiari G., Musarò A., Mattei M., Coviello T., Di Meo C., Matricardi P. (2021). Hyaluronan-Cholesterol Nanogels for the Enhancement of the Ocular Delivery of Therapeutics. Pharmaceutics.

