# Supplementary material for: Lyophilization for Formulation Optimization of Drug-Loaded Thermoresponsive Polyelectrolyte Complex Nanogels from Functionalized Hyaluronic Acid
Source: Pharmaceutics. 2023 Mar 13;15(3):929. doi: 10.3390/pharmaceutics15030929 (PMC10053597; doi:10.3390/pharmaceutics15030929)
Supplement: Supplementary file 1 [file pharmaceutics-15-00929-s001.zip › pharmaceutics-2256260-supplementary.pdf]

Supplementary Materials: Lyophilization for Formulation Optimization of Drug-Loaded Thermoresponsive Polyelectrolyte Complex Nanogels from Functionalized Hyaluronic Acid

Huu Van LE, Virginie DULONG, Luc PICTON, and Didier LE CERF *

1. Thermoresponsiveness of reconstituted PEC-NGs after lyophilization

HA-M2005/PLL PEC-NGs (n-/n+ = 2.5) prepared at C_P = 0.5 g.L^{-1} were lyophilized with 2 % m/v trehalose and then reconstituted at the same C_P in Milli-Q water. DLS characterization was performed on reconstituted PEC-NGs at 25 °C (after 120 s of temperature stabilization) and then at 50 °C (after 10 min of temperature stabilization) (**Error! Reference source not found.**).

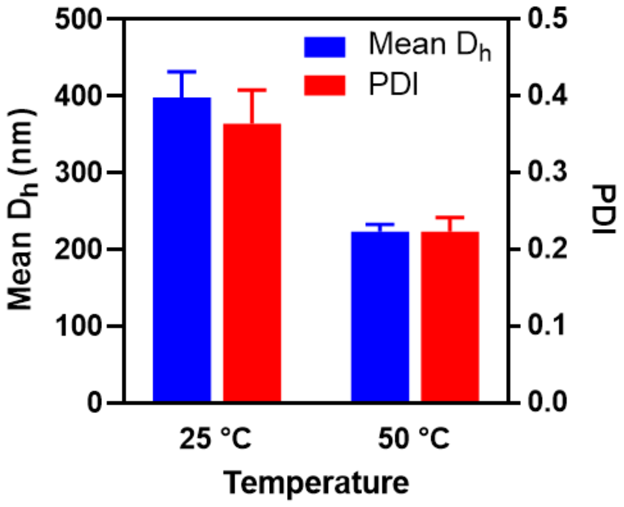

Figure S1. Mean D_h and PDI of reconstituted HA-M2005/PLL PEC-NGs (n-/n+ = 2.5, C_P = 0.5 g.L^{-1}) at 25 and 50 °C (n ≥ 3).

2. Stability of reconstituted PEC-NGs after lyophilization

HA-M2005/PLL PEC-NGs (n-/n+ = 2.5) prepared at C_P = 0.2 g.L^{-1} were lyophilized with 1 % m/v trehalose and then reconstituted in PBS to reach final C_P = 0.5 g.L^{-1}, thus C_{TRE} = 2.5 %. The reconstituted PEC-NGs were directly characterized by DLS (day 0) and then stored at 4 °C for revaluation after 1, 3 and 7 days (**Error! Reference source not found.**).

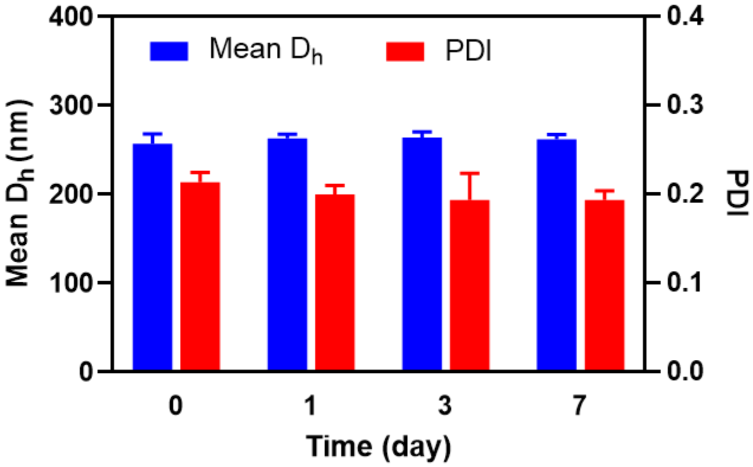

Figure S2. Mean D_h and PDI of HA-M2005/PLL PEC-NGs (n-/n+ = 2.5) reconstituted at C_P = 0.5 g.L^{-1} and C_{TRE} = 2.5 % m/v in PBS (after lyophilization of NGs prepared at C_P = 0.2 g.L^{-1}) within 7 days of conservation (n ≥ 3).

3. Model fitting of curcumin release data

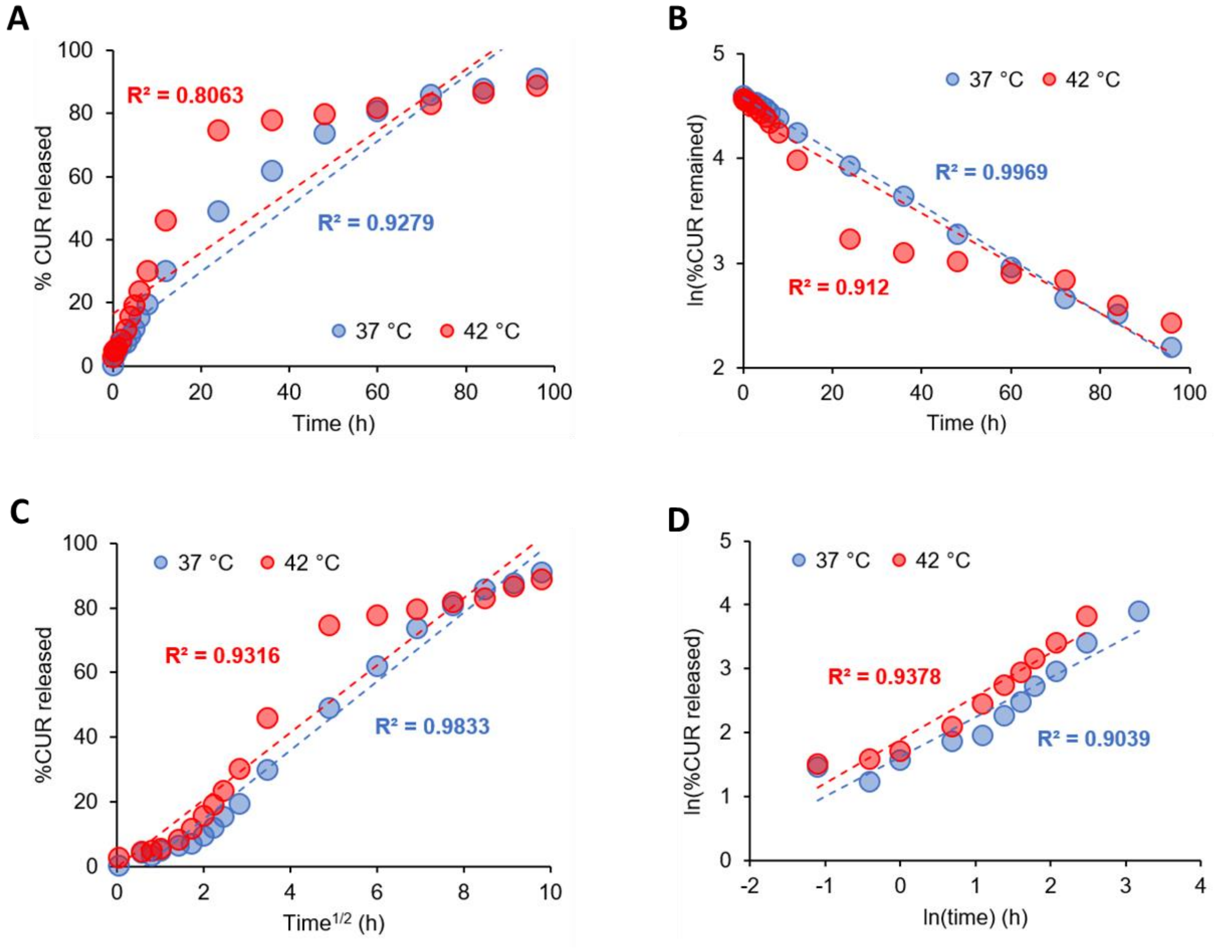

Figure S3. Mathematical model fitting of curcumin release data at 37 and 42 °C from HA-M2005/PLL PEC-NGs (n-/n+ = 2.5) prepared at C_P = 0.5 g.L^{-1}: (**A**) zero-order model (linear regression between time and CUR release fraction), (**B**) first-order model (linear regression between time and logarithm of CUR release fraction), (**C**) Higuchi model (linear regression between CUR release fraction and square root of time), (**D**) Korsmeyer-Peppas model (linear regression between logarithms of both time and the CUR release fraction within the first 60 %).
